# Gestational diabetes and macrosomia by race/ethnicity in Hawaii

**DOI:** 10.1186/1756-0500-6-395

**Published:** 2013-10-01

**Authors:** Pai-Jong Stacy Tsai, Emily Roberson, Timothy Dye

**Affiliations:** 1 John A Burns School of Medicine, University of Hawaii, 1319 Punahou Street, Suite 824, Honolulu, HI 96826, USA; 2 Hawaii Department of Health, 3652 Kilauea Avenue, Honolulu, HI 96816, USA

**Keywords:** Gestational diabetes, Ethnicity, Asian Pacific Islander, Macrosomia

## Abstract

**Background:**

Gestational diabetes (GDM) has been shown to have long-term sequelae for both the mother and infant. Women with GDM are at increased risk of macrosomia, which predisposes the infant to birth injuries. Previous studies noted increased rates of GDM in Asian and Pacific Islander (API) women; however, the rate of macrosomia in API women with GDM is unclear. The objective of this study was to examine the relationship between ethnicity, gestational diabetes (GDM), and macrosomia in Hawaii.

**Methods:**

A retrospective cohort study was performed using Hawaii Pregnancy Risk Assessment Monitoring System (PRAMS) data. Data from 2009–2011, linked with selected items from birth certificates, were used to examine GDM and macrosomia by ethnicity. SAS-callable SUDAAN 10.0 was used to generate odds ratios, point estimates and standard errors.

**Results:**

Data from 4735 respondents were weighted to represent all pregnancies resulting in live births in Hawaii from 2009–2011. The overall prevalence of GDM in Hawaii was 10.9%. The highest prevalence of GDM was in Filipina (13.1%) and Hawaiian/Pacific Islander (12.1%) women. The lowest prevalence was in white women (7.4%). Hawaiian/Pacific Islander, Filipina, and other Asian women all had an increased risk of GDM compared to white women using bivariate analysis. Adjusting for obesity, age, maternal nativity, and smoking, Asian Pacific Islander (API) women, which includes Hawaiian/Pacific Islander, Filipina, and other Asian women, had a 50% increased odds of having GDM compared to white women when compared using multivariate analysis. Among women with GDM, the highest prevalence of macrosomia was in white women (14.5%) while the lowest was in Filipina (5.3%) women.

**Conclusions:**

API women in Hawaii have increased rates of GDM compared to white women. Paradoxically, this elevated GDM risk in API women is not associated with an increased rate of macrosomia. This suggests the relationship between GDM and macrosomia is more complex in this population.

## Background

Gestational diabetes (GDM), defined as any degree of glucose intolerance with onset or first recognition during pregnancy, complicates approximately 7% of all pregnancies, resulting in more than 200,000 cases annually [1]. In the past decade, the overall incidence of GDM significantly increased even after adjusting for age and ethnicity [2]. GDM has been associated with adverse pregnancy outcomes, such as macrosomia, [3] birth trauma, [4] shoulder dystocia, [5] cesarean section, [3,6] pre-eclampsia, [7] preterm birth, [8] neonatal hypoglycemia, [3,4] and neonatal hyperbilrubinemia [4]. GDM predisposes women to a 3-fold increased risk of having infants with macrosomia, [9] which is an important risk factor for shoulder dystocia, [10,11] brachial plexus injury, [11,12] and perinatal mortality [12]. Women with macrosomic infants are at increased risk of cesarean section, hemorrhage, and perineal trauma [11].

Racial/ethnic differences in GDM have been noted in previous studies [13] and Asian and Pacific Islander (API) women were consistently shown to have the highest rates, ranging from 6 to 16%. A retrospective cohort study using birth certificate data in California noted GDM prevalence to be 10% among Asian women, higher than white (4.6%), Black (4.5%), and Hispanic (6.9%) women [14]. In a hospital-based cohort, Asian women had a 50 percent increased risk of GDM compared to white women [15]. Another study using birth certificate data in the U.S. showed API women had a substantially higher age-adjusted prevalence of GDM (6.3%) compared to white (3.8%), Black (3.5%), or Hispanic (3.6%) women. They also noted significant differences among API subgroups, ranging from 3.7% in Japanese women to 8.6% in Asian Indian women [16]. A study using self-reported data in Oregon noted non-Hispanic API women have the highest rates of GDM (14.8%) compared to Hispanic (11.1%), non-Hispanic Black (8.1%), non-Hispanic American Indian women (7.9%), and non-Hispanic Whites (6.0%). Within the API group, they found that Asian women had a higher prevalence of GDM (16.4%) compared to Pacific Islander women (11.7%) [17]. Using birth certificate data in New York, Chinese and non-Chinese East Asian/Pacific Islander women were found to have increased odds of GDM, OR 1.9 (95% CI 1.8-2.1) and 1.7 (95% CI 1.5-1.8), respectively, compared to white women [18].

Although studies clearly illustrated the increased risk of GDM in API women, perinatal outcomes associated with GDM, such as macrosomia, in API women are unclear.

Two studies showed a decreased risk of adverse perinatal outcomes in Asian women with GDM. Nguyen et al. [14] noted Asian women had lower odds of macrosomia, pre-eclampsia, cesarean section, neonatal hypoglycemia, and RDS when compared to white, black, and Hispanic women. Esakoff et al. [19] also noted decreased risk of macrosomia and cesarean section in Asian women with GDM compared to white women. In a hospital-based cohort of women with GDM, Silva et al. [20] noted an increased prevalence of macrosomia in neonates born to Hawaiians/Pacific Islander and Filipino women compared to neonates of Japanese, Chinese, Caucasian women.

Given this background, the objective of this study was to examine the relationship between ethnicity, particularly among Asians and Pacific Islanders, and the rates of gestational diabetes and macrosomia from a population perspective in Hawaii.

## Methods

This study was approved by the Western Institutional Review Board (IRB), the IRB of the Human Research Protection Office of the Centers for Disease Control and Prevention, and the State of Hawaii, Department of Health IRB. This study was conducted using Hawaii Pregnancy Risk Assessment Monitoring System (PRAMS) data from 2009 to 2011. PRAMS is a self-reported survey of recent mothers designed to collect information on maternal behaviors, attitudes, and experiences before, during, and immediately after pregnancy. PRAMS is a collaborative effort of the Centers for Disease Control and Prevention (CDC) and state health departments. Currently 40 states and New York City participate in the PRAMS program, representing 78% of all live births in the country. The PRAMS program uses a standardized data collection protocol including a mailed questionnaire (self-administered) with telephone follow-up for non-responders. Approximately 2,400 mothers a year in Hawaii are selected for participation as part of a stratified sample drawn from the certificates of live birth in Hawaii. Women complete the survey 3–9 months postpartum, with the majority responding at 3–4 months postpartum. The Hawaii PRAMS dataset includes information collected from PRAMS survey questions and from selected linked birth certificate variables. Data are weighted on an annual basis according to CDC protocol to be representative of all pregnancies resulting in live births in Hawaii in a given year. States must achieve a minimum response rate of 65% in order for survey results to be considered generalizable to all live births in the state in a given year. Hawaii PRAMS annual response rates have not fallen below 70% since data collection began in 2000, and the response rates for the years presented in this analysis ranged from 71-73%.

The following question from the PRAMS survey pertaining to gestational diabetes was used for this analysis: "During your most recent pregnancy, were you told by a doctor, nurse, or other health care worker that you had gestational diabetes (diabetes that started during this pregnancy)?" The independent variables included maternal age, maternal race/ethnicity, maternal pre-pregnancy body mass index (BMI), maternal nativity, household income, maternal education, marital status, smoking in the last 3 months of pregnancy, and presence of first trimester prenatal care. The dependent variables included presence of gestational diabetes and macrosomia (birth weight greater than 4000 grams) among women with gestational diabetes. Birth weight, along with maternal age, race/ethnicity, nativity, education, and marital status were determined based on linked birth certificate variables included in the Hawaii PRAMS dataset. Maternal race/ethnicity variables have been sorted into singly-coded groups using a standard algorithm used by the Hawaii Department of Health, Office of Health Status and Monitoring (OHSM) [21]. Maternal pre-pregnancy BMI, household income, smoking in the last 3 months of pregnancy, and presence of first trimester prenatal care were determined based on self-reported information in the Hawaii PRAMS survey.

Prevalence estimates and confidence intervals were generated for GDM and macrosomia using SAS-callable SUDAAN 10.0 to account for PRAMS’ complex sampling design. Descriptive statistics were generated to compare demographic information, including age, ethnicity, pre-pregnancy BMI, maternal nativity, household income, maternal education, marital status, smoking status, and first trimester prenatal care. For examining GDM rates, bivariate analysis was performed to identify potential confounders at p < 0.10. Multivariate logistic regression using forward selection was then performed to examine GDM and maternal ethnicity with whites as the referent group, controlling for BMI, age, maternal nativity, and smoking. Smoking was not significantly associated with GDM and maternal ethnicity, thus, was excluded from the final model. The final model adjusted for maternal age, BMI, and maternal nativity. Bivariate analysis was also used to examine the association between macrosomia and ethnicity in women with GDM. Due to limited cases of macrosomia in women with GDM by ethnicity, multivariate analysis was unable to be performed.

## Results

A total of 4735 participants that were weighted to represent all pregnancies resulting in live birth in Hawaii from 2009–2011 were evaluated. This cohort consisted of 22.8% white women, 38.1% Hawaiian/Pacific Islander women, 19.0% Filipina, and 14.9% other Asian women. Pacific Islander women included Samoan, Guamanian, and other Pacific Islanders. Other Asian women included Japanese, Chinese, Korean, Vietnamese, Asian Indian, and other Asian women. The breakdown of these groups was chosen based on their similarities with regards to other health outcomes observed in our population. The majority of participants received prenatal care in the first trimester of pregnancy and were above 25 years of age, of normal pre-pregnancy BMI, born in the U.S., college educated, married, and non-smokers in last 3 months of pregnancy. Maternal characteristics differed significantly by ethnicity (Table 1). Other Asian women had the highest percentage of advanced maternal age (33.8%) while Hawaiian/Pacific Islander women had the highest percentage of births to teens (12.5%). The highest prevalence of pre-pregnancy obesity was in Hawaiian/Pacific Islander women (23.8%), while the lowest was in other Asian women (7.4%). Over half (51.9%) of Filipinas were foreign-born. Hawaiian/Pacific Islander women had the highest prevalence of smoking in the last 3 months of pregnancy (11.1%), while the lowest was observed in Filipina women (4.3%).

**Table 1:** **Maternal characteristics by ethnicity**, **expressed in prevalence**

**Maternal characteristics**	**White N (%)**	**Hawaiian/PI**^**a **^**N (%)**	**Filipina N (%)**	**Other Asians**^**b **^**N (%)**	**P-value**
**Maternal age (yrs)**					
< 25	3340 (26.1)	9174 (44.0)	2667 (26.9)	1082 (11.7)	< 0.01
≥ 25	9473 (73.9)	11677 (56.0)	7255 (73.1)	8143 (88.3)	
**BMI**					
Underweight/Normal	8243 (66.2)	9754 (49.1)	5958 (63.0)	6554 (74.3)	< 0.01
Overweight/Obese	4217 (33.8)	10107 (50.9)	3506 (37.1)	2268 (25.7)	
**Maternal nativity**					
US born	11274 (88.0)	17316 (83.1)	4775 (48.1)	5945 (64.8)	< 0.01
Foreign born	1540 (12.0)	3535 (17.0)	5147 (51.9)	3224 (35.2)	
**Household income**					
< $10,000	703 (5.8)	6543 (33.7)	1541 (16.9)	755 (8.6)	< 0.01
$10,000-49,999	5791 (47.9)	8774 (45.2)	4508 (49.5)	3037 (34.6)	
≥ $50,000	5586 (46.2)	4098 (21.1)	3067 (33.7)	4996 (56.9)	
**Maternal education**					
High school or less	4665 (36.7)	13621 (66.8)	3983 (40.8)	2254 (24.8)	< 0.01
Some college	8037 (63.3)	6758 (33.2)	5769 (59.2)	6839 (75.2)	
**Married**					
No	2343 (18.3)	12360 (59.3)	4127 (41.6)	1832 (19.9)	< 0.01
Yes	10470 (81.7)	8492 (40.7)	5795 (58.4)	7393 (80.1)	
**Smoking last 3 months of pregnancy**					
No	11936 (94.7)	18223 (88.9)	9378 (95.7)	8552 (94.3)	< 0.01
Yes	669 (5.3)	2272 (11.1)	427 (4.3)	518 (5.7)	
**1 trimester prenatal care**					
No	1253 (10.0)	4908 (23.9)	1457 (15.0)	863 (9.4)	< 0.01
Yes	11347 (90.0)	15664 (76.1)	8228 (85.0)	8289 (90.6)	

^a^Includes Samoan, Guamanian, Other Pacific Islanders.

^b^Includes Japanese, Chinese, Korean, Vietnamese, Asian Indian, and other Asians.

The overall prevalence of GDM in Hawaii was 10.9%. GDM prevalence varied significantly by ethnicity, maternal age, BMI, nativity, and smoking in the last 3 months of pregnancy (Table 2). The increase in GDM was linear by maternal age for all ethnicities. Prevalence of GDM among women under 20 years of age was 4.6% while women above age 35 had a prevalence of 17.4%. The highest prevalence was in Filipina (13.1%) and Hawaiian/Pacific Islander (12.1%) women, while the lowest was in white women (7.4%). GDM prevalence was higher in women who were foreign-born, obese, and smoked in the last 3 months of pregnancy. The prevalence estimate for foreign-born women was 14.7% compared to 9.8% in U.S. born women. A linear increase in GDM prevalence by BMI was observed. Underweight women had a prevalence of 7.4% while obese women had a prevalence of 20.8%. A higher estimate of GDM was also noted in smokers (15.8%) versus non-smokers (11.1%). Hawaiian/Pacific Islander women, Filipinas, and other Asians all had increased odds of GDM compared to white women when compared using bivariate analysis (Table 2). After adjusting for obesity, age, and maternal nativity, API women had a 50% increased odds of GDM compared to white women (aOR 1.49, CI 1.05-2.11).

**Table 2:** Maternal characteristics of women with GDM

**Maternal characteristics**	**GDM (%)**	**GDM weighted N**	**P-Value**^**d**^	**Odds ratio (CI)**
Total sample	**10.9**	5925		
**Maternal ethnicity**				
White	**7.4**	934	**0.025**	Referent
Hawaiian/Pacific Islander^a^	**12.1**	2450	**0.002**	1.71 (1.19-2.44)
Filipina	**13.1**	1258		1.88 (1.26-2.79)
Other Asians^b^	**11.0**	989		1.53 (1.00-2.34)
White	**7.4**	934	**0.002**	Referent
Asian Pacific Islander^c^	**12.1**	4697		1.71 (1.22-2.38) aOR 1.49 (1.05-2.11)
**Maternal age (yrs)**				
< 25	**6.9**	1154	**<0.001**	Referent
≥ 25	**12.8**	4802		1.98 (1.45-2.70)
**BMI**				
Underweight/Normal	**6.8**	2127	**<0.001**	Referent
Overweight /Obese	**16.7**	3474		2.75 (2.13-3.55)
**Maternal nativity**				
US born	**9.8**	3972	**<0.001**	Referent
Foreign born	**14.7**	1985		1.59 (1.23-2.07)
**Household income**				
< $10,000	**9.5**	913	**0.462**	Referent
$10,000-49,999	**11.6**	2664		1.25 (0.88-1.79)
≥ $50,000	**11.2**	2050		1.21 (0.83-1.75)
**Maternal education**				
High school or less	**11.6**	2908	**0.201**	Referent
Some college or more	**10.1**	2867		0.85 (0.67-1.09)
**Married**				
No	**9.9**	2081	**0.151**	Referent
Yes	**11.7**	3876		1.20 (0.93-1.55)
**Smoking last 3 month of pregnancy**				
No	**10.6**	5330	**0.053**	Referent
Yes	**15.3**	620		1.51 (1.00-2.30)
**1st trimester prenatal care**				
No	**12.9**	1122	**0.154**	Referent
Yes	**10.5**	4712		0.80 (0.58-1.09)

^a^Includes Samoan, Guamanian, Other Pacific Islanders.

^b^Includes Japanese, Chinese, Korean, Vietnamese, Asian Indian, and other Asians.

^c^Includes Hawaiians, Other Pacific Islanders, Filipinos, and Other Asians.

^d^Data significance at p-value < 0.05.

**Figure 1:**
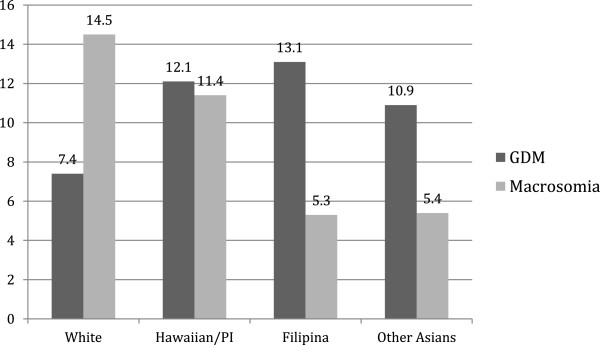
Prevalence (%) of GDM and associated macrosomia by ethnicity.

Estimates of GDM with associated macrosomia by ethnicity are shown in Figure 1. Among women with GDM, the highest prevalence of macrosomia was in white women (14.5%) while the lowest was in Filipina women (5.3%). The association between ethnicity and macrosomia in women with GDM was examined using bivariate analysis (Table 3). A trend towards increased odds of macrosomia and GDM compared to women without GDM was noted in all ethnic groups. These findings were not statistically significant and due to limited cases of macrosomia in women with GDM, multivariate analysis was unable to be performed.

**Table 3:** **Macrosomia** (**Birth weight** > **4000 g**) **among women with GDM compared to those without GDM**

	**GDM N (%)**	**No GDM N (%)**	**Bivariate OR**
**Maternal ethnicity**			
White	136 (14.5)	1458 (12.5)	1.19 (0.48-2.96)
Hawaiian/Pacific Islander^a^	280 (11.4)	1397 (7.8)	1.52 (0.84-2.75)
Filipina	67 (5.3)	145 (1.7)	3.18 (0.80-12.65)
Other Asians^b^	54 (5.4)	251 (3.1)	1.78 (0.43-7.35)
Asian and Pacific Islander^c^	401 (8.5)	1792 (5.2)	1.69 (1.02-2.80)

^a^Includes Samoan, Guamanian, Other Pacific Islanders.

^b^Includes Japanese, Chinese, Korean, Vietnamese, Asian Indian, and other Asians.

^c^Includes Hawaiians, Other Pacific Islanders, Filipinos, and Other Asians.

## Discussion

In Hawaii, API women have higher rates of GDM compared to white women. The overall GDM prevalence (10.9%) in Hawaii was higher compared to the national estimate (7%) [1], but the prevalence in API women (12.1%) was consistent with those reported in other parts of the country. Birth certificate data from California noted GDM prevalence to be 10% among Asian women, [14] while birth certificate data from Florida showed a GDM prevalence of 9.9% among API women [22]. Data from Oregon PRAMS observed that API had a GDM prevalence rate of 14.8% [17]. Thus, our data was consistent with previously published work.

One reason for the ethnic variation in GDM rates may be imperfect screening and a diagnostic process that over identifies glucose intolerance in certain racial/ethnic groups. GDM in the United States is generally diagnosed in a two-step process, although a one-step process has been suggested and is controversial. The two-step process includes a one-hour 50-gram glucose loading test (GLT) administered first. If the GLT test is above a certain threshold level, patients are then referred for a three-hour 100-gram glucose tolerance test (GTT). Diagnosis of GDM is made when two out of four values on the GTT are above a set threshold. Race-specific thresholds of these tests have been suggested in previous studies. For example, Nahum et al. [23] demonstrated that mean GLT values vary significantly by race. They found that Black women had the lowest mean GLT value (116.4 mg/dL) while Asian women had the highest (134.7 mg/dL). In this study, Asian women had the highest proportion (40.6%) exceeding the 140-mg/dL threshold, but the lowest proportion (11.5%) of positive GTT for women with the screening results greater than 140 mg/dL. Esakoff et al. [24] also showed different GLT test characteristics among different racial/ethnic groups. They found that to achieve 90% sensitivity and 10% false-positive rate, the optimal screening threshold was 135 mg/dL for Blacks, 140 mg/dL for whites and Latinas, and 145 mg/dL for Asians. Koklanaris et al. [25] cautioned against raising the screening threshold to 150 mg/dL for Asian women given the risk for the under-diagnosis of GDM. In their study of 95 Asian women with a GLT of 140–150 mg/dL, eight women (11.9%) were diagnosed with GDM. Although the authors found no differences in birth weight between the cases and controls (Asian women with GLT less than 140 mg/dL), they had concerns that raising the GLT threshold in Asian women may unacceptably lower sensitivity of the test. Ferrara et al. [26] examined the threshold values for GTT, and noted that regardless of the diagnostic thresholds used, the relationship of the GDM prevalence by ethnicity was similar. They compared two different GTT threshold cut-offs and found that although the overall GDM prevalence rate decreased with the use of one of them, Asian women still had the highest prevalence rate compared to other ethnicities. However, they failed to address whether the GTT thresholds should vary by ethnicity.

Not only does GDM prevalence differ by ethnicity, the interaction with obesity appears also to be affected by ethnicity. In a retrospective study using linked birth certificate and maternal hospital discharge data for live, singleton deliveries in Florida, ethnic differences in GDM were found to be attributable to being overweight and obese [22]. Only 15.1% of API GDM cases were attributable to being overweight and obese compared to 41.2% among non-Hispanic whites [22]. A similar retrospective study in California used a large managed care network and found that Asian and Filipina women had higher rates of GDM (9.9% and 8.5%, respectively) at a BMI of 22.0 to 24.9 kg/m^2^ compared to white, Black, and Hispanic women [27]. API women seem to be at risk of GDM at a lower BMI cut-off, but the association between GDM and obesity may not be as clear compared to that in other racial/ethnic groups.

In our study, the highest prevalence of macrosomia among women with GDM was in white women (14.5%) while the lowest was in Filipina women (5.3%). Women in all racial/ethnic groups had a trend towards increased odds of macrosomia in the presence of GDM compared to those without GDM. However, these findings did not reach statistical significance; likely due to the limited number of macrosomic cases for women with GDM. Lower rates of macrosomia in Asian women with GDM have been noted in previous studies. When examining racial differences in perinatal outcomes among women with GDM, Nguyen et al. [14] noted Asian women had lower odds of macrosomia compared to white, Black, or Hispanic women. Esakoff et al. [19] also noted decreased risk of macrosomia in Asian women with GDM compared to white women. In a hospital-based cohort of women with GDM, neonates born to Hawaiian/Pacific Islander and Filipino women had an increased prevalence of macrosomia compared to neonates of Japanese, Chinese, Caucasian women [20]. The racial/ethnic differences in macrosomia rates may be due to the inclusion of ethnicities in which a different definition of macrosomia should be applied. Generally, macrosomia is defined as birth weight greater than 4000 to 4500 grams or greater than 90th percentile. While this may be appropriate in homogeneous populations, it does not take into account physiologic variations among racial/ethnic groups that constitute populations within Hawaii. Many experts suggest using ethnicity-specific large-for-gestational age (LGA), defined as greater than the 90th percentile of birth weight within a particular race/ethnicity, which may be more appropriate for identifying high-risk infants. When surveying developing countries around the world, the World Health Organization (WHO) noted lower rates of macrosomia (birth weight greater than 4000 grams) in Asian countries, such as the Philippines (1.1%), India (0.5%), and Thailand (2.2%), compared to 14.9% in Algeria [28]. The cut-offs for 90th percentile birth weights were also lower in the Philippines (3485 grams), India (3250 grams), and Thailand (3630 grams) compared to a cut-off of 4050 grams in Algeria [28]. Even among macrosomic infants, there are racial/ethnic disparities in perinatal complications. In a study from New Zealand, infants with birth weights greater than 4500 grams born to Asian mothers were compared to those born to Polynesian mothers [29]. The infants born to Asian mothers were more likely to be admitted to the neonatal intensive care unit, require intravenous dextrose, and have respiratory distress. To address this issue, countries such as the UK, Canada, New Zealand, and Australia have adopted the use of customized growth curves which account for maternal ethnicity to identify high-risk babies [30-35]. A study conducted in Canada found that approximately 61 per 1000 male and 57 per 1000 female LGA newborns of South Asian descent would be missed if conventional rather than ethnicity-specific birth weight curves were used [31]. In a prospective study in New Zealand and Australia, the use of customized birth weight curves showed a stronger association with adverse outcomes compared to conventional birth weight curves [35]. In ethnically diverse regions in the U.S., such as Hawaii, the use of customized, ethnicity-specific growth curves should be implemented to correctly identify at-risk infants of GDM mothers.

Our study’s sample was a strength, representing all live births in Hawaii. However, this study is not without limitations. One such limitation is that Hawaii PRAMS survey data is self-reported, and consequently subject to bias due to recall or reporting factors. Additionally, the self-reported data do not allow for confirmation of the clinical diagnosis of GDM, or for further analysis of clinical data such as degree of glycemic control, lifestyle modification, and medication compliance. Another limitation was the need to collapse multiple ethnic groups into four larger groups due to small numbers. For example, the Other Asian women group includes diverse groups of people such as Chinese, Filipino, Laotian, Hmong, Korean, Japanese, and Vietnamese, into a single large group. We recognize the diversity within all of the racial/ethnic groups in our study. Unfortunately, aggregation was done to overcome limited sample size in our study. Also, as a result of the OHSM race algorithm used in Hawaii, API women in our sample could not be identified as mixed race/ethnicity. Finally, our unique population in Hawaii may not be generalizable to other populations given the high numbers of women of mixed race and ethnicity.

## Conclusions

Similar to other regions of the country, Asian and Pacific Islander women in Hawaii have increased rates of GDM compared to white women. Paradoxically, this elevated GDM risk in API women was not associated with increased rates of macrosomia. This suggests that the relationship between GDM and macrosomia is probably more complex than generally appreciated, and that a redefinition of "macrosomia" should be considered. Thus, the use of customized growth curves to identify LGA infants may be more appropriate in an ethnically diverse population such as that of Hawaii. Further research on ethnicity-specific diagnosis in GDM and macrosomia is needed. More importantly, interpreting the associations between these conditions and perinatal complications among diverse populations will be a vital step towards improving pregnancy outcomes.

## Abbreviations

GDM: Gestational diabetes; API: Asian Pacific Islander; PRAMS: Pregnancy Risk Assessment Monitoring System; CDC: Center for Disease Control and Prevention; IRB: Institutional Review Board; BMI: Body mass index; GLT: Glucose loading test; GTT: Glucose tolerance test; LGA: Large for gestational age; SGA: Small for gestational age.

## Competing interests

The authors declare that they have no competing interests.

## Authors’ contributions

PT made substantial contributions to the conception and design of the study, analysis and interpretation of data, and draft the manuscript. ER made substantial contribution to the acquisition of data, analysis and interpretation of data, and been involved in the revision of the manuscript. TD made substantial contribution to the conception and design of the study, analysis and interpretation of data, and been critically involved in the revision of the manuscript. All authors read and approved the final manuscript.
